# Challenges and opportunities in remote sensing-based crop monitoring: a review

**DOI:** 10.1093/nsr/nwac290

**Published:** 2022-12-19

**Authors:** Bingfang Wu, Miao Zhang, Hongwei Zeng, Fuyou Tian, Andries B Potgieter, Xingli Qin, Nana Yan, Sheng Chang, Yan Zhao, Qinghan Dong, Vijendra Boken, Dmitry Plotnikov, Huadong Guo, Fangming Wu, Hang Zhao, Bart Deronde, Laurent Tits, Evgeny Loupian

**Affiliations:** State Key Laboratory of Remote Sensing Science, Aerospace Information Research Institute, Chinese Academy of Sciences, Beijing 100101, China; School of Resources and Environment, University of Chinese Academy of Sciences, Beijing 100049, China; Executive Committee of Group on Earth Observations Global Agricultural Monitoring (GEOGLAM), Geneva 2300, Switzerland; State Key Laboratory of Remote Sensing Science, Aerospace Information Research Institute, Chinese Academy of Sciences, Beijing 100101, China; Executive Committee of Group on Earth Observations Global Agricultural Monitoring (GEOGLAM), Geneva 2300, Switzerland; State Key Laboratory of Remote Sensing Science, Aerospace Information Research Institute, Chinese Academy of Sciences, Beijing 100101, China; School of Resources and Environment, University of Chinese Academy of Sciences, Beijing 100049, China; Executive Committee of Group on Earth Observations Global Agricultural Monitoring (GEOGLAM), Geneva 2300, Switzerland; State Key Laboratory of Remote Sensing Science, Aerospace Information Research Institute, Chinese Academy of Sciences, Beijing 100101, China; Queensland Alliance for Agriculture and Food Innovation, The University of Queensland, Brisbane 4343, Australia; State Key Laboratory of Remote Sensing Science, Aerospace Information Research Institute, Chinese Academy of Sciences, Beijing 100101, China; State Key Laboratory of Remote Sensing Science, Aerospace Information Research Institute, Chinese Academy of Sciences, Beijing 100101, China; State Key Laboratory of Remote Sensing Science, Aerospace Information Research Institute, Chinese Academy of Sciences, Beijing 100101, China; Queensland Alliance for Agriculture and Food Innovation, The University of Queensland, Brisbane 4343, Australia; Department of Remote Sensing, Flemish Institute of Technological Research, Mol 2400, Belgium; Department of Geography and Earth Science, University of Nebraska-Kearney, NE 68849, USA; Department of Satellite Monitoring Technologies, Space Research Institute of Russian Academy of Sciences, Moscow 117997, Russia; State Key Laboratory of Remote Sensing Science, Aerospace Information Research Institute, Chinese Academy of Sciences, Beijing 100101, China; School of Resources and Environment, University of Chinese Academy of Sciences, Beijing 100049, China; State Key Laboratory of Remote Sensing Science, Aerospace Information Research Institute, Chinese Academy of Sciences, Beijing 100101, China; State Key Laboratory of Remote Sensing Science, Aerospace Information Research Institute, Chinese Academy of Sciences, Beijing 100101, China; School of Resources and Environment, University of Chinese Academy of Sciences, Beijing 100049, China; Department of Remote Sensing, Flemish Institute of Technological Research, Mol 2400, Belgium; Department of Remote Sensing, Flemish Institute of Technological Research, Mol 2400, Belgium; Department of Satellite Monitoring Technologies, Space Research Institute of Russian Academy of Sciences, Moscow 117997, Russia

**Keywords:** crop monitoring, crop condition, crop production, Ground data, Remote Sensing

## Abstract

Building a more resilient food system for sustainable development and reducing uncertainty in global food markets both require concurrent and near-real-time and reliable crop information for decision making. Satellite-driven crop monitoring has become a main method to derive crop information at local, regional, and global scales by revealing the spatial and temporal dimensions of crop growth status and production. However, there is a lack of quantitative, objective, and robust methods to ensure the reliability of crop information, which reduces the applicability of crop monitoring and leads to uncertain and undesirable consequences. In this paper, we review recent progress in crop monitoring and identify the challenges and opportunities in future efforts. We find that satellite-derived metrics do not fully capture determinants of crop production and do not quantitatively interpret crop growth status; the latter can be advanced by integrating effective satellite-derived metrics and new onboard sensors. We have identified that ground data accessibility and the negative effects of knowledge-based analyses are two essential issues in crop monitoring that reduce the applicability of crop monitoring for decisions on food security. Crowdsourcing is one solution to overcome the restrictions of ground-truth data accessibility. We argue that user participation in the complete process of crop monitoring could improve the reliability of crop information. Encouraging users to obtain crop information from multiple sources could prevent unconscious biases. Finally, there is a need to avoid conflicts of interest in publishing publicly available crop information.

## Teaser

The paper identifies the challenges and opportunities of crop monitoring with remote sensing. In-situ data accessibility and the negative effects of knowledge-based analysis are essential to reduce the applicability of crop monitoring.

## INTRODUCTION

The establishment of a more resilient food system for the realization of the United Nations Sustainable Development Goals is reliant on various factors, including the collection of concurrent and near-real-time data for monitoring agricultural production conditions and infrastructures to allow rapid and accurate data processing [1,2]. Uncertainties in global food markets have dramatically increased recently due to the adversely changing climate, catastrophic events (such as droughts, floods, the COVID-19 pandemic, desert locusts, and regional conflicts), and increasingly strained global competition and trade. Therefore, it is critical to establish efficient and inclusive data collection and analysis systems to provide the necessary information for agricultural policy-making [2] and to maximize the impacts of policy interventions with limited resources. Furthermore, accurate, near-real-time information affects the entire agricultural production chain, and obtaining this information could enhance the capabilities of the importers and/or exporters of agricultural products in advance of negotiations, allowing them to better cope with the substantial fluctuations in global food prices that have become evident during the current food logistics disruption resulting from the COVID-19 pandemic and regional conflicts. Such information can also aid in improving farm management support for farmers, securing the informed hiring of seasonal employees, adjusting pricing schemes for traders or insurance companies, altering stock and logistics routes for suppliers, revising national food balance sheets to guide food imports and exports, and mobilizing food aid for humanitarian purposes [3].

Satellite remote sensing has become one of the major methods used for local, regional and global crop monitoring since the 1970s [4]. Here, the term ‘crop monitoring’ specifically refers to monitoring for staple crops rather than the general term of agricultural monitoring, which includes livestock, horticulture and aquaculture. In general, a typical crop monitoring activity consists of agroclimatic analyses, crop condition and stress monitoring, and crop production predictions, some systems also include food security assessments and thus an early warning of likely food insecurities. From the perspective of remote sensing, crop monitoring mainly focuses on crop growth status and ultimate production. Agro-climate analysis and food security assessment are the analysis of its causes and impacts.

Currently, free access to medium- to high-resolution satellite data has generated new opportunities in the development of timely and all-weather satellite-driven crop monitoring capacities at high spatial and temporal resolutions [5,6]. Cloud-computing platforms, such as Amazon, Google Earth Engine (GEE) and Microsoft AI for Earth, have greatly enhanced the capabilities of satellite data processing and information extraction techniques, together with meteorological, soil and elevation information and other auxiliary data [7]. Thus, satellite data and processing capacities no longer serve as constraints in crop monitoring.

Therefore, the focus of crop monitoring has shifted to developing new methods for retrieving crop information from satellite-derived data and establishing robust crop monitoring systems (CMSs) [6,8–10]. However, combinations of crops, locations, geographic extents and temporal dynamics make obtaining crop monitoring data streams a complex task. Most recently developed methods have failed to be implemented as operational activities (Table 1). In addition, nine CMSs with global and/or regional operational perspectives exist, but only a few have full functions. Most systems mainly target crop conditions and do not have crop yield or production prediction capabilities [8]. Few national CMSs are currently in full operation and publicly accessible globally. Most national CMSs still rely on traditional field surveys to derive crop acreage or to obtain average yield estimates with the support of remote sensing products such as land cover or crop data layer (CDL) [11]. Progress in crop monitoring has not fundamentally changed the methods of obtaining necessary information for agricultural policy-making or altered pathways by which agricultural statistics are derived, despite the sharp increase in the availability of Earth observation data [7] and the development of national EO programmes both in developed and developing countries.

**Table 1.** Methods used in existing CMSs.

System	Coverage	AgroClimate	Crop condition		Crop production			Independent drought	Food security	Reference
			Status	Stress analysis	Crop types	Crop area	Yield	monitor		
ASIS [30]	Global	P profile & P departure from average; Accumulative P and profiles	NDVI anomaly and profiles and VCI	ASI, weighted VHI over Gaul2 and mean VHI	Cropland and grassland masks	n.a.	n.a.	ASI, drought intensity and frequency	CCBS	https://www.fao.org/giews/
China CAS & CAAS										
CropWatch [31]	Global	P, T, PAR and potential biomass anomalies over 15 years and profiles	NDVI anomaly and VCIx over the last five years, NDVI development and clustering	VHI, flooding, diseases and pests	Grains, wheat, maize, rice and soybean	Remote sensing-based crop type mapping; CPTP	Agro-meteo, RS index, Biomass + harvest index	SPI, VCI, TCI, VHI, NDWI, and soil moisture	Supply trend	http://cropwatch.cn
CHARMS	China	Anomaly maps and profiles	NDVI anomaly maps and development	n.a.	Wheat, maize, rice, and soybean	Areal sampling and crop classification	RS index WOFOST model	NDDI, TVDI, and anomaly of actual ET	n.a.	Personnel communication, offline
European Union										
ASAP [32]	Global	SPI1, SPI3, and GWSI	zNDVIc and mNDVId	SPI, GWSI and zNDVIc anomalies	Crop and rangeland masks	n.a.	Work in progress [33]	GWSI, NDVI, and automatic drought warning	CAF threshold	https://mars.jrc.ec.europa.eu/asap/
MARS [26]	Europe & neighbours	SPEI, ASI, WOFOST, and PET	VCI, VPI, and CNDVI	AOC maps and warning index	MARS crops	From EUROSTAT with a specific calendar	CoBo & BioMa	WSI and precipitation anomaly	n.a.	
USDA NASS, FAS/IPAD & USAID										
Crop Explorer	Global	AgroClimate for Crop Explorer	NDVI departure from average, previous year and previous decade	Soil moisture and T thresholds for particular crops	CADRE crops	Unknown	Crop water production functions from CADRE	SPI, P and ET anomalies, heat damage and stress	Balance sheet	https://ipad.fas.usda.gov/cropexplorer/
NASS, VegScape	USA	P and T departures from normal	NDVI, VCI, RVCI, MVCI, and RMVCI	n.a.	Wheat, corn, soybeans, cotton, and potatoes	June area survey with CDL	Monthly objective yield survey	n.a.	n.a.	https://nassgeodata.gmu.edu/VegScape
FEWS-NET	30 countries	Rainfall assumptions: average and accumulative	NDVI and NDVI anomaly (%) with 2001–2018 mean, accumulative values and 8-day time series		n.a.	n.a.		WRSI, VHI, and P anomaly	SD and IPC	https://fews.net/
GLAM [34]	Global	P, T and ET departures from normal		ESI, actual ET, SMI, and SWI anomalies	Cultivated cropland mask	n.a.	n.a.	NDWI, SWI, and P anomaly	n.a.	https://glam.nasaharvest.org/
Crop Monitor [35]	Global	Anomalies of P and T sums			Crop-specific masks	n.a.	n.a.	n.a.		https://cropmonitor.org/
WFP Seasonal Explorer	Global	P accumulation, anomalies and ranking since 1981	NDVI percentage average; development of NDVI and average	n.a.	Cropland and rangeland mask	n.a.	n.a.	P anomaly, NDVI percentage average and T ranking	n.a.	https://dataviz.vam.wfp.org/seasonal_explorer/reports
OZ-wheat [36–40]	Australia	n.a.	n.a.	n.a.	Wheat, sorghum	n.a.	Crop stress index model	Simulated crop stress with meteorological data	n.a.	
AAFS [36–40]	Australia	Seasonal P & T and their comparisons to average; P percentiles	NDVI anomaly	VHI provided by FAO ASIS	Up to 158 commodities	From ABS	Statistical forecasting methods	RSMP	Balance sheets	
PAK-SCMS	Pakistan	Monthly P v. previous year, maximum and minimum T v. last two years	NDVI, anomaly maps and profiles	Water supply, pests, and nitrogen	Rice, wheat, cotton, sugarcane	Crop classification	Remote sensing-based statistical model	Anomaly of precipitation; water supply situation	n.a.	https://suparco.gov.pk/crop-management/
FASAL	India	Anomaly map	VI anomaly map and development	n.a.	Rice, wheat, potato, rapeseed/mustard	Crop classification with *in situ* samples	Remote sensing-based statistical model	n.a.	n.a.	
VEGA [41]	Russia	Maps and profiles of cumulated P	NDVI anomaly map and development, MVCI, RVCI, and NDVI normalized on GDD	n.a.	Cropland, winter crops, summer crops, clean fallows	Remote sensing-based crop type mapping	Remote sensing-based statistical model	Comparison with cumulated average precipitation	n.a.	http://vega.geoglam.ru//?lang=eng
CALMS [42]	Canada	Agro-climatic models	NDVI anomaly map and development	Soil moisture and anomaly maps	Spring wheat, barley, canola	Crop classification with *in situ* samples	Statistical forecast with NDVI, WDI, and GDD	SM and SM anomaly	n.a.	

Abbreviations: AAFS = Australian Agricultural Forecasting System; ABS = Australia Bureau of Statistics; AgroClimate for Crop Explorer = % of normal P at the 5-day, weekly and monthly scales, average, maximum and minimum T and departure from normal, extreme maximum and minimum T, snow depth and cover; AOC = areas of concern indicating excessive or deficit rain, radiation deficit, heatwave, temperature accumulation surplus or deficit, and fAPAR; ASAP = anomaly hot spots of agricultural production; ASI = Agricultural Stress Index; ASIS = Agricultural Stress Index System of the Food and Agriculture Organization of the United Nations (FAO); BioMA = crop growth modelling platform incorporated with WOFOST, CropSYS, STICS, CaneGro for sugarcane and WARM for rice; CAAS = Chinese Academy of Agricultural Sciences; CADRE crops = wheat, rice, and coarse grains (corn, barley, sorghum, and oats), oilseeds (soybeans, rapeseed, and palm), and cotton; CADRE = Crop Assessment Data Retrieval & Evaluation; CAF = critical area fraction; CALMS = Canadian Ag-Land Monitoring System; CAS = Chinese Academy of Sciences; CCBS = country cereal balance sheet; CDL = cropland data layers; CHARMS = China agriculture remote sensing monitoring system; CNDVI = accumulated NDVI from the start of the growing season; CoBo = control board with different statistical methods to produce yield forecasts; CPTP = crop-planting proportion and crop type proportion method; ESI = Evaporative Stress Index; ET = evapotranspiration; FAS = Foreign Agricultural Service; FASAL = forecasting agricultural output using space, agrometeorological and land based observations of India; FEWS-NET = famine early warning systems network; GDD = growing degree days above 5°C; GLAM = global agricultural monitoring; GWSI = global water requirement satisfaction index; IPC = Integrated Food Security Phase Classification; MARS = monitoring agricultural resources; MARS Crops = wheat, barley, maize, rye, triticale, rapeseed, sugar beet, potato, sunflower, and soybean; mNDVId = mean of the difference between NDVI and its long-term average over the growing season; MVCI = relative change in NDVI compared to the mean NDVI; NASS = National Agriculture Statistics Service; NDVI = normalized difference vegetation index; NDWI = Normalized Difference Water Index; P = precipitation; PAK-SCMS = Pakistan satellite-based crop monitoring system; PASG = % of average seasonal greenness; PET = potential evapotranspiration; RMVCI = relative change in NDVI compared to the median NDVI; RSMP = Relative Soil Moisture Percentiles; RVCI = Relative NDVI change compared to the previous year; SD = Scenario Development for Food Security Early Warning; SMI = soil moisture index; SPI = standard precipitation index; SWI = soil water index; T = temperature; USDA = United States Department of Agriculture; VCI = vegetation condition index; VHI = vegetation health index; VPI = vegetation productivity indicator; WDI = water deficit index; WFP = UN World Food Programme; WRSI = crop water requirement satisfaction index; zNDVIc = standardized anomaly of cumulative NDVI over the growing season.

Recent reviews in the field of crop monitoring [6,12,13] reveal a substantial body of research on topics of crop mapping [14,15], crop condition assessments [16–18], crop yield predictions and forecasting [3,19,20], drought monitoring [21–23], CMSs [8,9] and precision agriculture [24,25]. They summarize popular topics in research and are dedicated to technical methods, but seldom discuss what has limited crop monitoring activities in providing reliable and actionable information in support of food security. Few raise the concern that the lack of reliable crop information poses a serious threat for decision making with respect to food security. Therefore, in this paper, we review recent developments in crop monitoring while mainly focusing on limitations in the operational capacities of these methods. Then, we discuss the consequences of these limitations and propose solutions for bridging some of these constraints to improve and strengthen the reliability and applicability of crop monitoring information in the context of food security decision making.

## DERIVING CROP CONDITIONS AND STRESS

Temporal-spatial and qualitative information on crop conditions is critical to policy-making, preventing market disruption and speculation, thereby contributing to early warning of food security [26]. Such attributes are the main focus of crop monitoring (Table 1) [8,27]. Crop biophysical features are considered proxies of crop conditions. Multispectral vegetation indices (VIs) or metrics, which are relevant to the morphological, physiological and biophysical traits of crops, have been developed to describe the growth status of crops. These include the normalized difference vegetation index (NDVI), crop canopy cover, leaf area index (LAI), fraction of absorbed photosynthetically active radiation (fAPAR), aboveground biomass (AGB), net primary productivity (NPP), leaf chlorophyll content (LCC), canopy chlorophyll content (CCC), solar-induced chlorophyll fluorescence (SIF), leaf equivalent water thickness (LEWT), crop water deficit, vegetation health index (VHI) and growth stage [28,29]. These metrics have been applied to assess crop growth status and the impacts of agroclimatic conditions, pests and diseases, water stress and management practices on crop growth and, hence, to support early warning systems.

Sudden changes in abnormal weather conditions could result in stressed crops, and the negative impacts are complicated, intertwined and often associated with specific crops, growing stages, and genetic varieties. For instance, the significantly below-average wheat conditions observed in Central Europe in 2016 were mainly a result of insufficient PAR [43]. Heat stress is critical, especially for wheat between the flowering and grain-filling stages, when the maximum temperatures exceed 32°C [44,45]. Frost damage and stress caused by low temperatures and/or cold waves are also commonly observed in winter crop cultivation regions after the jointing stage, although winter crops are more tolerant to low temperatures than are summer crops [46]. However, indicators and/or metrics are mainly applied for assessing crop conditions, stress and drought severity, which can then be used to infer variations in yield, area and production in qualitative categorized methods and criteria. These assessments fail to quantitatively convert indicators and/or metrics into crop growth status [47,48] and unmix the stresses and disturbances. Thus, further studies need to consider the intersystem consistency and community awareness that the comparability of crop information across CMSs is low due to different categorized criteria of crop conditions. Standard and quantitative methods for monitoring crop growth status and stress should be explored, which need to incorporate more biophysically and biochemically related VIs to investigate the causes of stress by abiotic factors such as floods [49] and drought [22] and/or biotic factors due to diseases and pests. To identify and explain what drives crop stress, the integration and synthetic analysis of multiple factors, including climatic (precipitation, temperature, radiation, etc.), environmental (soil moisture), biophysical (LAI) and biochemical (nitrogen content) variables, are needed.

Hyperspectral data and light detection and ranging (LiDAR) data in combination with optical data are promising in effectively improving the capability to output early warnings and discriminate the causes of regional crop stress [50] and the severity of the stress in terms of effects on final yield in a quantitative way. However, those satellites are currently unable to support operational requirements due to narrow swaths, large footprints or low resolutions.

### Limitations on monitoring crop conditions

There are similar methods employed across global, regional and national CMSs for analysing crop conditions in near-real-time, most of which rely on maps of anomalies of metrics from the average values to investigate spatial variations, or on temporal development to reflect crop growth dynamics (Table 1). These methods require a seamless and comparative historical archive of metrics and a real-time satellite data processing capacity to produce biophysical products using dedicated algorithms. The differences are then qualitatively interpreted as crop growth classes. There are three ways to present these metric differences: (1) an anomaly map at a specific date, indicating spatial variations and offering comparisons across large regions; (2) aggregated profiles of current and reference years to reflect the development of crops over the growing season for the specific spatial extent, as derived from the VI time series, showing the start, length, ascending and descending slope, and peak of crop greenness; and (3) spatial clustering maps in which pixels reflecting similar crop development conditions are grouped [31].

However, the differential method cannot provide reliable assessments due to a lack of standard categorized methods and criteria and may be biased due to shifts in crop phenology, crop rotations and other factors [29,51]. Aligning the time series VI curves according to the accumulated growing degree days based on the air temperature or the results of in-season crop growth modelling could thus avoid the occurrence of misleading information caused by crop phenology shifts and could suppress biased information and improve the overall crop condition monitoring performance [52]. The maximum vegetation condition index (VCIx) [31] over the crop growing season, for example, over a three-month period, can generate spatially consistent crop condition series by eliminating phenological impacts arising from large latitude spans. Additionally, the proportion of land that is cultivated with crops may vary greatly across different years, thus limiting the comparability of vegetation signals. The integration of cropped versus uncropped arable land ratios derived from high-spatial-resolution data and coarse-resolution VI time series could generate adjusted VI data and reduce the uncertainties caused by temporal crop rotation shifts and spatial shifts in the cropped area [29].

In terms of indicators, although many metrics could potentially be used or are already in use by some national, regional, or global CMSs, the most used variable is NDVI, which has a strong relationship with the crop AGB, leaf area, light interception, and yield and offers relatively simple operation and timeliness [9,53]. However, NDVI is insensitive to densely vegetated areas and sensitive to soil background variations. Nevertheless, the high NDVI values caused by excessive crop growth do not necessarily represent favourable conditions for crop production [26]. Other indicators such as EVI, LAI, AGB, NPP and fAPAR are rarely used due to their crop-specific uncertainties, complex processing methods and the need for ancillary and baseline data, although they have been verified to be less affected by signal saturation and highly effective for various crop conditions [54]. Alternatively, multiple indices can be combined to overcome the limitations of individual indices [31] and to take full advantage of multisource satellite data, including optical and synthetic aperture radar (SAR) data, to develop temporally comparable crop condition indices for different phenological periods, thus reflecting the real crop conditions and alleviating signal saturation bias [55].

Identifying a proper baseline product and a suitable remote sensing product at a suitable spatial resolution could effectively reduce uncertainties in crop condition monitoring. For instance, as crop conditions vary significantly between irrigated and rainfed crops, especially during the dry seasons (and in dry-prone regions), these conditions can be monitored individually according to location-specific irrigation conditions [56]. The current crop condition monitoring methods still largely use low-resolution satellite data (with spatial resolutions ranging from 250 m to 1 km) [8,26]; these data often contain multiple crops in coarse pixels and can rarely indicate the conditions of individual crops, except in large parcels. The emerging availability of Sentinel-2–like satellite data makes medium- to high-resolution crop condition monitoring possible, though invariably with a vast amount of data processing, while high spatial-resolution data could also lead to other issues, such as geolocation mismatch and soil background impacts [57]. Users should be free to select appropriate spatial scale data for their specific monitoring targets.

### Limitations on detecting crop stress driven by drought

Drought is the major natural disaster that causes the most extensive crop stress and yield losses [58], and drought assessments are incorporated in most CMSs as part of the crop condition component or individual component (Table 1). A lack of precipitation combined with higher evaporation rates can propagate from a meteorological drought into an agricultural drought, leading to a reduction in crop yield or even to complete crop failures. In this regard, many drought indices have been developed for detecting meteorological droughts caused by climate variabilities, such as the standardized precipitation index (SPI), the standardized precipitation evapotranspiration index (SPEI) and the Palmer drought severity index (PDSI).

Drought conditions directly affect the morphology, greenness, photosynthesis, biomass accumulation, and evapotranspiration of crops. Many vegetation-based agricultural drought indices have been developed, such as the vegetation condition index (VCI) and mean vegetation condition index (MVCI). Given that drought events cause soil moisture drying and land surface temperature (LST) changes, the temperature condition index (TCI) based on LSTs, soil moisture agricultural drought index (SMADI) [22,59], evaporative stress index (ESI) and hydrothermal weather index (HWI) have been proposed to determine agricultural drought conditions. The LST is a timely response indicator that can reflect crop stress before substantial visual symptoms arise [23]. The popular VHI is the combination of VCI and TCI reflecting both biophysical and environmental conditions.

However, some of the indices developed for detecting drought compensate for the difference between meteorological drought and agricultural drought by incorporating climate variables, environmental variables and the vegetation status (Table 2). When monitoring a meteorological drought, indicators derived from climatic variables, such as SPI, are sufficient to reflect climatic anomalies. Adding variables such as soil moisture, which might be affected by anthropogenic anti-drought measures, reduces the effectiveness of using climate variables alone. An actual agricultural drought may not occur if agricultural practices are appropriately implemented to preserve soil water moisture in accordance with crop-specific water requirements, even after a severe meteorological drought. Therefore, agricultural drought indicators cannot be straightforwardly derived from climate variables or validated with climate variables; for example, it would be incorrect to use SPI-3 and/or SPEI-3 to verify agricultural drought indices.

**Table 2.** Summary of typical studies conducted using multivariate analyses to develop drought indicators, including the input variables and validation methods.

	Climatic variable		Environmental variable			
Indicator	Precipitation	Evaporation	Soil moisture	Soil temperature	Vegetation status	Validation method
ESI [62]		ET, PET		MODIS LST	LAI	*In situ* yield
IDMI [63]	PCI		SMCI		VCI	SPI
VegDRI [64]	SPI, PDSI				PASG, NDVI	USDM
OMDI, OVDI [65]	TRMM		AMSR-E SM, CLSMAS	MODIS LST	NDVI	SPEI
GIIDI [66]	PCI		SMCI	TCI	VCI	*In situ* PDSI, SPI, SPEI, and Z-index
SDCI [67]	TRMM			MODIS LST	NDVI	*In situ* PDSI and Z-index
MIDI [68]	PCI		SMCI	TCI		*In situ* SPI, SPEI, and EVI
DSI [69]		ET, PET			NDVI	Drought-damaged crop area and yield statistics
PADI [69]	PCI		SMCI		VCI	SPI, PDSI, and yield loss
PMDI	PCI		SMCI	TCI	VCI	SPEI, NDVI, and SIF

Abbreviations: AMSR-E = Advanced Microwave Scanning Radiometer-EOS; CLSMAS = China Land Surface Soil Moisture Assimilation System; DSI = drought severity index; ESI = evaporative stress index; GIIDI = geographically independent integrated drought index; IDMI = integrated drought monitoring index; ISDI = integrated surface drought index; LAI = leaf area index; MIDI = microwave integrated drought index; MODIS = moderate resolution imaging spectroradiometer; OMDI = optimized meteorological drought index; OVDI = optimized vegetation drought index; PADI = process-based accumulated drought index; PASG = percent of average seasonal greenness; PCI = precipitation condition index; PDSI = Palmer drought severity index; PMDI = meteorological drought index by PCA-RF method; SDCI = scaled drought condition index; SDI = synthesized drought index; SIF = solar-induced chlorophyll fluorescence; SM = soil moisture; SMCI = soil moisture condition index; SPEI = standardized precipitation evapotranpiration index; TCI = temperature condition index; TRMM = Tropical Rainfall Measuring Mission; USDM = U.S. Drought Monitor; VCI = vegetation condition index; VegDRI = vegetation drought response index; Z-index = Palmer moisture anomaly index.

Because drought is a recurrent phenomenon that is highly associated with climate variabilities [60], many indices involve the relative differences of indicators, such as the departure of the indicators from their long-term averages, to qualitatively reflect the impacts of drought; these indices include the precipitation condition index (PCI), soil moisture condition index (SMCI), VCI, TCI, and many other indices [61]. All of these indices are normalized over a given reference period. However, there is also a lack of any standardized method for converting the differential values of indices into drought severity information.

### Limitations on determining the impacts of nutrients, diseases and pests on crop stress

If crop stress is not caused by adverse weather, it is likely to be caused by nutrient stress, diseases or pests. Nutrients have been reported as another stress factor after water stress at the global scale [70]. Inversion algorithms involving CCC or leaf nitrogen content (LNC) have been developed to detect nutrient stress in wheat and other crops [25]. Although many visible-band VIs have been developed to relate to chlorophyll or nitrogen content, red-edge bands, special bands located between the red and near-infrared bands, have been proven to be more sensitive to chlorophyll content [71]. The advantage of the red-edge band in detecting the nutrient status or chlorophyll content has been continuously demonstrated [72], but it is only effective for dense crops. Sentinel-2 satellites have three red-edge bands, making it possible to detect chlorophyll content using imagery from these satellites.

Many metrics have been developed to identify types of diseases and pests, assess the corresponding infection severities, and map their distributions at the plot or regional scale [18]. However, prior knowledge is needed to identify the types of local disease/pest or other stresses that occur in the field. In areas for which prior knowledge is lacking, it is thus difficult to proactively achieve reliable and precise assessment [18], as a variety of signs and plant damage caused by crop diseases and pests can also be caused by other factors, such as nutrition deficiencies, thus leading to challenges when attempting to separate the actual stress factor. For instance, the photochemical reflectance index (PRI) is used not only for wheat yellow rust detection, but has also commonly been used to detect water stress, frost stress and damage, and nitrogen content and stress [73]. New indicators and metrics are needed to distinguish the various causes of stress and to quantify different severities.

Nevertheless, hyperspectral data are more advanced in detecting leaf biochemical constituents or identifying abnormal spectral features affected by pests and diseases through spectral derivatives, continuous removal transformations and continuous wavelet transformations. The scattering from a leaf responds differently at different wavelengths to changes in leaf properties such as pigment concentration, other chemical constituents [74], or disturbances of pests or diseases. A blueshift was observed in the red-edge peak in the first derivative of rice plant spectral curves infested by rice leaf folder [75], as well as in the PRI for detecting wheat yellow rust [76] and the chlorophyll absorption ratio index (CARI) for wheat powdery mildew [77]. However, the low temporal resolution of satellite hyperspectral sensors due to narrow swaths makes it infeasible to implement them in operational activities. Therefore, the detection of nitrogen stress or the identification of crop diseases and pests are rarely implemented in operational crop monitoring. Alternatively, new sensors with narrow spectral bands, which are sensitive to nitrogen stress, diseases and pests, and wide swaths are expected to increase temporal resolution.

## FORECASTING CROP PRODUCTION

Crop production is forecast with the support of crop area estimates and yield predictions for specific combinations of agro-ecological regions, administrative units and crop types. Crop type mapping and geostatistical methods are two categories of methods to derive crop area estimations, while crop type mapping not only provides data to estimate crop area, but also provides baseline data for crop condition assessment and yield prediction [31].

### Limitations on crop type mapping

Most crop-mapping studies have been conducted in local areas with high dependence on field data and lack transferability to other regions [6]. Additionally, most methods rely heavily on local knowledge of management practices, phenology and prior knowledge of cropping patterns [14]. Thus, crop area estimates are constrained by the spatial and temporal representativeness of the *in situ* data used for training the classifiers [10]. Although high-resolution satellite data provide rich spectral and textural information, crop mapping methods are relatively well developed only for local areas, with an overall accuracy (OA) of approximately 66%–94%, but with a lower accuracy of only 50%–79% at early growing stages, while mapping crops to a larger extent remains a challenge [10]. Accurate classification models calibrated for one region cannot be readily extrapolated to another region due to the location specificity of crop phenotype and phenology information or the differences in canopy-level spectral reflectance among different environments and management practices [5]. In addition, irregularities in the data acquisition time and cloud-cover conditions throughout the crop-growing season make it even more challenging to develop a universal crop-mapping method incorporating only optical remote sensing data. To overcome such limitations, a series of methods locally calibrated with *in situ* data collected in a crowdsourcing manner [78] by various partners across China and regional characteristics have been developed to identify staple crops, including winter wheat, canola, rice, soybean and maize, at 10-m resolution over the major agricultural production regions of China with a relatively high accuracy of up to 97% [79].

**Figure 1.**
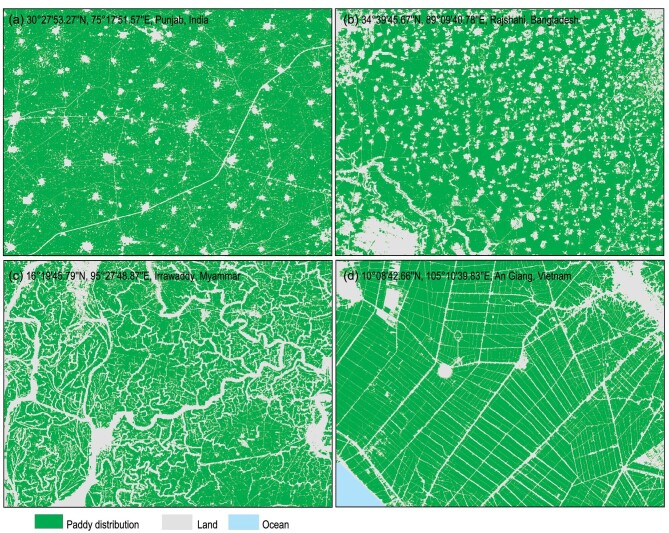
a–d, paddy rice in Southeast and South Asia at 10 m resolution in 2020.

Methods for exploiting all-weather imaging SAR data to eliminate cloud impacts in optical data have increased in popularity in crop areas where these data are available. SAR has already been used to identify paddy rice fields [80]. The coupling of the interferometric and backscattering information of SAR data can significantly improve crop type mapping. The first 10-m–resolution crop type map based on the Copernicus Sentinel-1 and Sentinel-2 satellites was successfully produced for the entire European Union (EU) [81] and paddy rice of China [82], with respective OAs of 76% for 19 crop types and 90% for paddy rice, underpinning the operational delivery of in-season, high-resolution crop maps from the local scale to the global scale. The rice mapping algorithm was also extended to South and Southeast Asia, generating a 10-m–resolution paddy rice map for 2020 (Fig. 1).

Machine learning and deep learning techniques are widely used in crop mapping but achieve different performances, with OAs ranging from 70% to 99% [13,83]. More models, frames or complex architectures are expected to exploit the spectral and temporal dimensions of satellite data and fuse other features extracted by various methods to improve the overall performance. Therefore, the incorporation of multisensor data, satellite-derived crop phenology, and cropping practice information [83,84], as well as the application of both spatial (e.g. field-measured data) and historical data (e.g. crop rotation histories) [85] to deep learning methods, constitute major future directions that can lead to cloud services to ensure globally accurate crop mapping and area estimation results. Furthermore, transfer learning methods, in which a model pretrained on a large dataset could be easily fine-tuned to improve the prediction effect on the target dataset, must be further developed to improve the learning performance and reduce the reliance on *in situ* data, thus reducing the dependence on ground data collection efforts, which need to couple crop phenology and prior knowledge of cropping practices.

When the crop type map is determined, the crop area can be estimated, as it is an intrinsic derivative from the map. However, the crop area cannot be estimated directly by counting pixels in crop maps, as the classification errors and resolution bias will directly affect the resulting crop area statistics. Resolution bias is unavoidable and depends on the spatial resolution and fragmentation of the agricultural landscape [86]. Pixels can cover small ponds, canals, and other noncrop features, thus resulting in resolution bias, particularly in mountainous areas. Thus, the actual crop area in a given region must be adjusted using an unbiased estimator by measuring the effects of the mixture of arable land pixels [87]. Such an unbiased estimator could be derived by overlapping high-resolution crop field delineation based on AI technologies and targeted arable land masks over selected sample plots (Table 3).

**Table 3.** Unbiased estimator to adjust crop area estimation.

Provinces of China	Location	Morphology	Field size (ha)	Unbiased estimator
Heilongjiang	126.72°E, 45.40°N	Plain	10	0.894
Hebei	114.91°E, 37.02°N	Plain	1.0	0.814
Inner Mongolia	108.47°E, 40.96°N	Slope	0.5	0.875
Jiangsu	120.19°E, 33.20°N	River network	0.2	0.742
Sichuan	105.47°E, 28.82°N	Terrain	0.1	0.599

### Limitations on geostatistical methods

Geostatistical methods to derive crop areas generally rely on field survey information based on area sampling frames with the support of satellite data products [88,89]. Early attempts obtained cropped areas by extrapolating selected subregion crop mapping to a larger region with statistical inference [4,89]. However, large uncertainties and time lags in crop mapping make these methods infeasible for crop area estimation. In contrast, cropped and noncropped arable lands can be easily separated or segmented using remote sensing due to the contrast signals of crops with respect to the land surface. Therefore, the crop-planting proportion, as estimated by segmenting cropped and noncropped areas from satellite data, and the crop type proportion, as estimated by transect sampling results, were multiplied by the arable land area to obtain the cropped areas with a relative error of approximately 4% at the crop strata, provincial and national levels [89]. Freely accessible, full-coverage public satellite data help to derive the crop-planting proportion during the growing season. This method to derive seasonal crop acreage estimations for regions with complex agricultural practices [89], while a crop type mapping method is applied over regions with homogeneous agricultural landscapes, was called CPTP in CropWatch [27]. However, both require field data to quantify the crop type proportions or to train the classification algorithms. A complete area estimation also requires skills in crop mapping, spatial sampling and a survey of the sample sites [33]. These requirements constitute the primary factor preventing many CMSs from containing crop area estimation components (Table 1).

### Limitations on predicting crop yield

**Figure 2.**
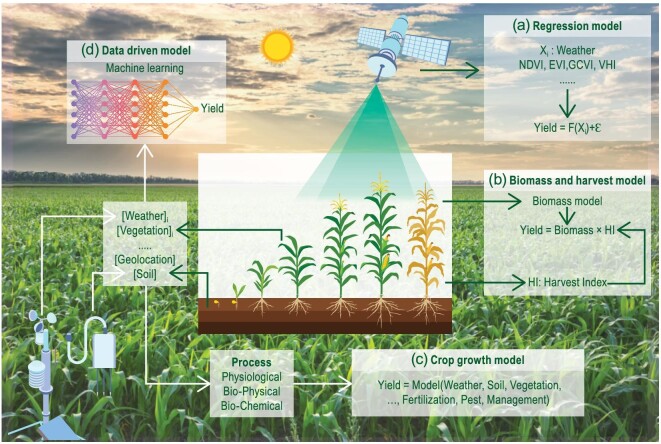
Crop yield prediction methods. a, regression method; b, biomass and harvest index; c, crop growth model; d, data-driven models.

At present, four types of satellite-driven methods have been developed for predicting crop yields weeks or months ahead of harvest, including (a) statistical regression methods, (b) crop growth models, (c) biomass and harvest indices, and (d) machine learning methods (Fig. 2). Methods are calibrated and transformed into a final crop yield to forecast food production. Most food security programs use approaches that combine satellite data with agroclimate indices, which are calibrated and transformed into a final crop yield to predict food production.

- Statistical regression methods use remote sensing VIs, metrics (the same as crop conditions) and/or weather variables that are specific to the targeted locations and time spans. Weather variables correlated well with crop yield in rainfed agricultural regions, as weather conditions drive crop growth and crop stresses [90]. However, it is clear that statistical models do not fully reflect plant stresses due to complicated soil–plant–atmosphere interactions and can only capture 67%–80% of yield variance [3,90]. Incorporation of VIs can improve the model ability, with an increase in the coefficient of determination (*R*^2^) of approximately 2%–10% [91]. However, VI saturation in dense crop canopies can lead to poor index performance when predicting crop yields [57]. The integration of optical and SAR data can help predict crop yields by reducing the saturation effects of VIs [92]. The VH and VV backscattering coefficients and VH:VV ratio of the SAR are related to the water content and geometric information of the crop canopy. The interferometric coherence and VH:VV ratio are sensitive to the canopy development of wheat [93]. The coherence SAR information coupled with the red-edge vegetation index also performs well for rice yield prediction at the heading stage [94]. The integration of weather variables, remote sensing-based VIs and other metrics, such as the SIF values from the Global Ozone Monitoring Experiment version 2 (GOME-2), allowed more accurate crop yield prediction [95]. The Sentinel-2–based red-edge chlorophyll index combined with the soil-regulated vegetation index achieved optimal results in plot-scale yield prediction [54].
  While the regression models are simple and straightforward, these models often require recalibration using new field measurements for new locations. Models using both remote sensing and weather data may suffer from collinearity issues among the exogenous input variables [3]. The accuracy of yield prediction increases with the development of the crop growth stage [90]. However, each new setup requires a proper validation step. In the future, more satellite data could be incorporated, but inevitably, this will create some collinearity issues and as such will need to be validated across temporal and spatial scales.
- Grain formation is mainly influenced by two factors: (1) the accumulation of aboveground dry matter, i.e. the crop biomass, and (2) the proportion of dry matter that is converted to grains, i.e. the crop harvest index [31]. The crop harvest index varies over time and among cultivars and is a determining factor in the yield formation process. Most VIs showed strong correlations with crop yields until anthesis or until peak biomass accumulation, after which their predictive power diminished [3], with reported *R*^2^ values in the range of 0.69–0.79 for estimating the harvest index with remote sensing [96]. This finding suggests that the estimation of harvest indices from satellite data needs to be further explored.
- Crop growth models integrate remote sensing indicators and weather variables by data assimilation to better accommodate changes in the location, weather, and timing of satellite data. More specifically, models could capture crop growth patterns more accurately by incorporating time-sequential remotely sensed metrics [95] to generate the spatial distributions of crop yields [97]. However, the strong uncertainty of canopy state variables and soil properties derived from satellite data significantly reduces the accuracy of crop yield prediction. In addition, only a limited number of remote sensing-derived variables, mostly crop emergence dates and LAI, have been incorporated into crop models, and these variables differ somewhat from the agronomic variables [6]. Pixel-based remote sensing-derived metrics such as the LAI are often average values of complex agricultural landscapes, but the LAI variable in a crop model assumes a homogeneous land surface. The complexity of crop physiological processes posed a difficulty to accurate simulation by crop growth models, resulting in great variations of the yield forecasting accuracy across the growing stages and regions, ranging from 0.3 to 0.97 [90,98,99], and hindered the crop model when scaling the results up for use in operational processes. One alternative is to train empirical models by correlating the model outputs and other variables, including remotely sensed metrics, with actual yields to improve their predictive power [100]; this would require the support of highly accurate ground observation data or production data at the administrative unit, but this still captures only approximately one-third of the field yield variation [101].
- As many factors influence crop yields, machine learning and deep learning methods have recently been extensively explored to forecast crop yields [19,20]. Machine learning or deep learning-based methods establish statistical relationships among VIs, weather variables, soil proportions and crop yields to predict crop yield [102]. The selection of statistical algorithms, feature engineering and data processing strategies exerts a strong impact on crop yield prediction ability. Random forests (RFs) [54], support vector machines (SVMs) [33], artificial neural networks (ANNs), convolutional neural networks (CNNs), long short-term memory (LSTM) [103] and deep neural networks (DNNs) are the most widely used algorithms, with *R*^2^ values between 0.15 and 0.78 [19,20]. Due to the diversity of the machine learning algorithms, models with climate data alone (*R*^2^ of 0.59–0.73) sometimes outperform models with VIs alone (*R*^2^ of 0.49–0.70) [104]. This suggests that the appropriate algorithms and feature engineering should be carefully compared and designed to improve the overall performance.

Overall, the yield prediction component is the weakest component in crop monitoring due to large uncertainties. This also indicates that current models and/or VIs cannot fully comprehend the determinants of crop yields, especially under extreme climatic conditions [3]. For example, wheat yields in France declined significantly in 2016 due to unusual extreme climate conditions involving abnormally warm temperatures in late autumn 2015 and unusually wet conditions in the following spring; however, the MARS system in Europe failed to predict wheat yields correctly for this year [105]. This was mainly due to the inaccurate crop yield models for extreme climate conditions, as the observed extreme climate and environmental conditions had rarely occurred before. VI saturation also leads to poor performance of these indices when predicting yields, especially in dense or irrigated crop regions. In addition, the uncertainty of current crop growth models makes it difficult to scale these models up to facilitate operational yield predictions. CropWatch adopts averaged values of three yield models to reduce the uncertainty of yield prediction [31]. Furthermore, new sensors need to be explored for predicting crop yields, specifically from the perspective of geometric structures with simultaneous observations in the optical, SAR and thermal infrared bands in narrow spectra and multiview azimuth angles; this would allow the phenotypic characteristics related to the physiological processes of photocatalysts to be measured more accurately at the canopy, field and regional levels.

## CONSEQUENCES AND SOLUTIONS OF THE LIMITATIONS IN CROP MONITORING

Limitations in current crop monitoring methods have been identified in the previous sections. Some limitations, including *in situ* data accessibility and knowledge-based analysis, might reduce the applicability of crop monitoring and lead to uncertain and undesirable consequences.

Existing methods usually require new ground-truth data for each new setting to parameterize algorithms and models and assess their accuracies. The field sampling requirements prevent most global systems from obtaining crop area estimates and yield prediction components (Table 1), as collaboration with local institutions is required to conduct field work [26] and access to *in situ* data for training and calibrating algorithms and modelling outside national boundaries is still challenging. GEOGLAM has implemented an *in situ* data coordination strategy to leverage partner investments and to ensure that data are curated with a standard protocol. GEOGLAM embraces the Global Earth Observation System of Systems (GEOSS) Data Sharing Principles that encourage full open sharing of data, including both EO data and *in situ* data, although open sharing of *in situ* data is still a challenge, as it sometimes involves privacy issues. Nevertheless, the GEOGLAM Joint Experiment for Crop Assessment and Monitoring (JECAM) initiative demonstrates a best practice method of data sharing to enhance the availability of *in situ* data through intercomparison projects or scientific data papers [10,106,107]. However, the ground-truth dataset is not yet fully publicly available due to restrictions imposed by the in situ providers. Moreover, in the foreseen future, it is unrealistic to expect the full sharing of ground-truth data with increasing trade tensions and strained global cooperation. Therefore, the requirement for ground-truth data in crop monitoring should encourage crop monitoring activities at the domestic and local levels.

**Figure 3.**
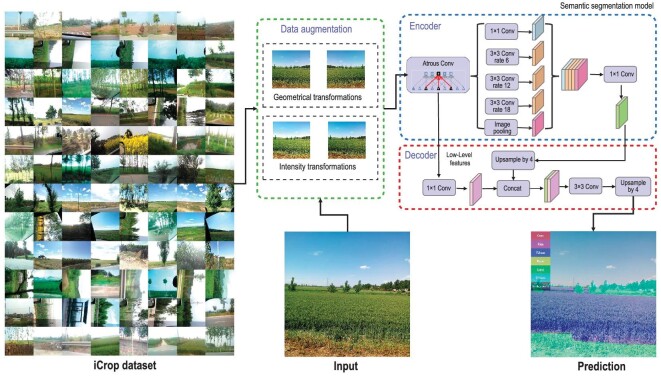
Automatic crop type identification from geo-tagged photos using a deep learning algorithm.

As in situ data collection is one of the major challenges for crop monitoring, closing the ground-truth data gaps and improving the data collection efficiency are essential for strengthening the reliability of crop monitoring. However, the acquisition of field data, especially at large scales, is time- and cost-consuming and labour-intensive. To address this issue, crowdsourcing might provide an alternative and efficient solution for acquiring field-based data [78]. Crowdsourcing information has become a widespread data acquisition method in environmental and resource monitoring [108], serving as a potential solution for closing the ground-truth data gaps. With the wide use of mobile phones, smartphone sensors, such as cameras, satellite positioning, and photoreceptors, have become major platforms for crowdsourcing information collection [109]. A mobile global positioning system (GPS)-video-geographic information systems (GIS) application (called a GVG app) can collect such data as crop types, planting dates, irrigation and expected yields with corresponding geolocation information [78]. Convolutional neural networks (CNNs) have been used to automatically identify crop types from Google Street photos or GVG photos (Fig. 3) [78,110].

**Figure 4.**
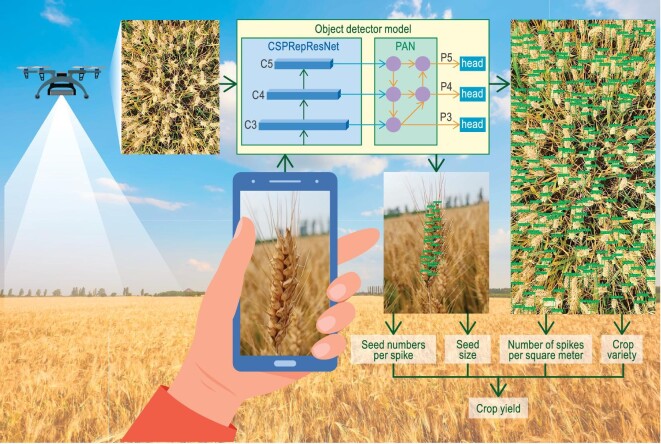
Framework for nondestructive yield data measurements.

Data collection of the actual crop yield is not only labour-intensive and costly, but also difficult to be implemented efficiently. It relies on the grain harvest of samples in the field with uncertainties in both sampling and unavoidable grain losses during harvest. A new method for field yield data measurement involving AI and computer vision to count the numbers of spikes, seed numbers per spike and the sizes of seeds for weight determination (Fig. 4) is urgently needed for integration into GVG.

The reliability of crop information is essential, as such information serves as an important resource factor with significant economic value and consequences. There is a lack of transparent and standardized methods for synthesizing various information in crop monitoring to support decision making [47], thus affecting food prices [111]. Instead, knowledge-based analyses are mostly applied in crop monitoring activities, especially in the process of generating actionable reports. Analysts explore the indicators provided by the system and identify the indicators that best explain the actual crop growth and crop stress conditions. They then select robust methods to conduct accurate crop acreage estimates, yield predictions, and production forecasts for specific agroclimatic regions and publish the results in the form of regular bulletins. Therefore, analysts must specialize in the specific region for which they have expertise in regional agroclimatic conditions and management practices if they are to understand how the crop indicators generated by the system describe the actual yield variations in that region. In this case, the personal knowledge, views, or preferences of analysts all affect their working practices.

To ensure the reliability of a synthesized crop monitoring report, it needs to follow a transparent and interactive process with respect to users. Although satellite images have neutral aesthetics because of their photogenic quality, their production and analysis have profound political [112] and economic implications. Crop monitoring of global/regional CMSs provides free access to crop information, but publicly released crop information corresponds with obvious business interests with significant economic value and consequences. Clearly, by establishing global and regional CMSs and releasing crop information regularly, hosts have ensured their dominant position in food export opportunities and enhanced their voices in global food security governance. Thus, there are concerns that these CMSs are more likely to fail to disseminate crop monitoring information that may be unfavourable to the hosts [112]. Additionally, it would be extremely difficult to question these reports without direct access to the algorithm code and the underlying data used to generate such information. Therefore, it is essential that potential conflicts of interest are avoided to maintain the integrity of unbiased crop information released by those global or regional crop monitoring activities. For example, crop monitoring should be conducted by an entity entirely independent of government or business bodies, or by an international agency.

To mitigate these subjective effects, the GEOGLAM Crop Monitor performs a collective negotiation process to confirm the derived results by consulting local partners, but limits the involvement of analysts in the generation of the input data products. The international analysis team of CropWatch is facilitated through a platform that enables analysts who specialize in specific regions to call indicator data and thematic maps to assist in the analyses and work jointly to output bulletin chapters and sections. This participatory approach ensures the transparency of the analytical process and provides expert knowledge as a reference for the generated reports in supporting decision making for stakeholders.

Alternatively, crop monitoring should be inclusive of users and provide user-driven services. All components and functions of CropWatch, including the self-calibration abilities of models and the collaborative analyses of indicators, were transferred to APIs in the CropWatch-Cloud, which enables users to carry out self-serviced crop monitoring by selecting their preferred indicators for the user's area of interest. This allows users to complete crop monitoring independently and autonomously from the data download to the final synthesized analysis. For example, with the support of a customized CropWatch for Mozambique local conditions, officials in the Mozambique Ministry of Agriculture and Rural Development (MARD) who respond to crop monitoring and earlier warming can apply specific programming language environments to call APIs and organize processing workflows [1] and can also set up self-defined projects/systems for any areas of interest in their country by invoking the appropriate APIs. As users from MARD have defined the modules themselves, calibrated and used the tools, MARD enhances the capability and reliability of crop monitoring for Mozambique without additional investment in storage and computational resources. This effort was recognized as one of the best rural solutions in 2020 by the International Fund for Agricultural Development and one of the good practices in South–South and Triangular Cooperation for Sustainable Development.

Furthermore, it would be better for users to obtain crop information from their own systems or from different sources to ensure the reliability and representativeness of information and to prevent unconscious biases. This is why, immediately after the global food crisis of 2008, the Group of Twenty (G20) Agriculture Ministers launched a crop monitoring initiative with international participation, i.e. GEOGLAM during the French G20 Presidency in 2011. The objectives of GEOGLAM were to increase market transparency, improve food security and stabilize commodity prices by producing and disseminating crop information and enhancing crop monitoring capacities. The dissemination of global or regional crop information from various hosts, including CropWatch (Table 1), increases the availability and transparency of food-related information by providing regularly released bulletins and reports.

## CONCLUSION

Satellite-derived crop monitoring approaches have been utilized by various institutes and agencies to inform policymakers about issues related to crop production and food security, but they are still far from providing near-real-time, reliable and quantitative crop information, although satellite data and processing capacities are no longer constraints. In this paper, we reviewed the systematic progress made to date, the likely limitations, and the future development pathways and proposed potential solutions to address the existing issues in crop monitoring efforts. We have identified that *in situ* data accessibility and knowledge-based analysis of satellite-derived metrics are two essential issues that reduce the applicability of crop monitoring and lead to undesirable consequences. There is a need to explore satellite data to better capture determinants of crop production and to enhance analytical capacities in order to transform satellite-derived metrics to understandable and useful knowledge for stakeholders. In particular, there is a need to be concerned about conflicts of interest when publishing publicly available crop information.
